# Supplementary material for: Burden and bacterial etiology of neonatal meningitis at Hawassa University Comprehensive Specialized Hospital, Hawassa, Ethiopia
Source: PLoS One. 2024 Aug 9;19(8):e0308523. doi: 10.1371/journal.pone.0308523 (PMC11315337; doi:10.1371/journal.pone.0308523)
Supplement: Attachment — Submitted filename: Response to Reviewers.docx [file pone.0308523.s002.docx]

PONE-D-24-15982

Burden and etiology of neonatal meningitis at Hawassa University Comprehensive Specialized Hospital

PLOS ONE

Dear Dr. Ali,

Thank you for submitting your manuscript to PLOS ONE. After careful consideration, we feel that it has merit but does not fully meet PLOS ONE’s publication criteria as it currently stands. Therefore, we invite you to submit a revised version of the manuscript that addresses the points raised during the review process.

==============================

Please submit your revised manuscript by Jul 20 2024 11:59PM. If you will need more time than this to complete your revisions, please reply to this message or contact the journal office at [plosone@plos.org](mailto:plosone@plos.org). When you're ready to submit your revision, log on to https://www.editorialmanager.com/pone/ and select the 'Submissions Needing Revision' folder to locate your manuscript file.

Please include the following items when submitting your revised manuscript:

- A rebuttal letter that responds to each point raised by the academic editor and reviewer(s). You should upload this letter as a separate file labeled 'Response to Reviewers'.
- A marked-up copy of your manuscript that highlights changes made to the original version. You should upload this as a separate file labeled 'Revised Manuscript with Track Changes'.
- An unmarked version of your revised paper without tracked changes. You should upload this as a separate file labeled 'Manuscript'.

If you would like to make changes to your financial disclosure, please include your updated statement in your cover letter. Guidelines for resubmitting your figure files are available below the reviewer comments at the end of this letter.

If applicable, we recommend that you deposit your laboratory protocols in protocols.io to enhance the reproducibility of your results. Protocols.io assigns your protocol its own identifier (DOI) so that it can be cited independently in the future. For instructions see: <https://journals.plos.org/plosone/s/submission-guidelines#loc-laboratory-protocols>. Additionally, PLOS ONE offers an option for publishing peer-reviewed Lab Protocol articles, which describe protocols hosted on protocols.io. Read more information on sharing protocols at <https://plos.org/protocols?utm_medium=editorial-email&utm_source=authorletters&utm_campaign=protocols>.

We look forward to receiving your revised manuscript.

Kind regards,

Tebelay Dilnessa, MSc
Academic Editor
PLOS ONE

Journal requirements:

When submitting your revision, we need you to address these additional requirements.

1. Please ensure that your manuscript meets PLOS ONE's style requirements, including those for file naming. The PLOS ONE style templates can be found at

https://journals.plos.org/plosone/s/file?id=wjVg/PLOSOne_formatting_sample_main_body.pdf and

<https://journals.plos.org/plosone/s/file?id=ba62/PLOSOne_formatting_sample_title_authors_affiliations.pdf>

**Response:** I have checked and corrected accordingly.

2. We note that your Data Availability Statement is currently as follows: [Author declares that there is no competing interest.]

Please confirm at this time whether or not your submission contains all raw data required to replicate the results of your study. Authors must share the “minimal data set” for their submission. PLOS defines the minimal data set to consist of the data required to replicate all study findings reported in the article, as well as related metadata and methods (https://journals.plos.org/plosone/s/data-availability#loc-minimal-data-set-definition).

For example, authors should submit the following data:

- The values behind the means, standard deviations and other measures reported;

- The values used to build graphs;

- The points extracted from images for analysis.

Authors do not need to submit their entire data set if only a portion of the data was used in the reported study.

If your submission does not contain these data, please either upload them as Supporting Information files or deposit them to a stable, public repository and provide us with the relevant URLs, DOIs, or accession numbers. For a list of recommended repositories, please see https://journals.plos.org/plosone/s/recommended-repositories.

If there are ethical or legal restrictions on sharing a de-identified data set, please explain them in detail (e.g., data contain potentially sensitive information, data are owned by a third-party organization, etc.) and who has imposed them (e.g., an ethics committee). Please also provide contact information for a data access committee, ethics committee, or other institutional body to which data requests may be sent. If data are owned by a third party, please indicate how others may request data access.

**Response**: I have uploaded dataset as supplement file.

3. Your ethics statement should only appear in the Methods section of your manuscript. If your ethics statement is written in any section besides the Methods, please move it to the Methods section and delete it from any other section. Please ensure that your ethics statement is included in your manuscript, as the ethics statement entered into the online submission form will not be published alongside your manuscript.

4. We note that Figure 1 in your submission contain [map/satellite] images which may be copyrighted. All PLOS content is published under the Creative Commons Attribution License (CC BY 4.0), which means that the manuscript, images, and Supporting Information files will be freely available online, and any third party is permitted to access, download, copy, distribute, and use these materials in any way, even commercially, with proper attribution. For these reasons, we cannot publish previously copyrighted maps or satellite images created using proprietary data, such as Google software (Google Maps, Street View, and Earth). For more information, see our copyright guidelines: http://journals.plos.org/plosone/s/licenses-and-copyright.

We require you to either (1) present written permission from the copyright holder to publish these figures specifically under the CC BY 4.0 license, or (2) remove the figures from your submission:

a. You may seek permission from the original copyright holder of Figure 1 to publish the content specifically under the CC BY 4.0 license.

We recommend that you contact the original copyright holder with the Content Permission Form (http://journals.plos.org/plosone/s/file?id=7c09/content-permission-form.pdf) and the following text:

“I request permission for the open-access journal PLOS ONE to publish XXX under the Creative Commons Attribution License (CCAL) CC BY 4.0 (http://creativecommons.org/licenses/by/4.0/). Please be aware that this license allows unrestricted use and distribution, even commercially, by third parties. Please reply and provide explicit written permission to publish XXX under a CC BY license and complete the attached form.”

Please upload the completed Content Permission Form or other proof of granted permissions as an "Other" file with your submission.

In the figure caption of the copyrighted figure, please include the following text: “Reprinted from [ref] under a CC BY license, with permission from [name of publisher], original copyright [original copyright year].”

b. If you are unable to obtain permission from the original copyright holder to publish these figures under the CC BY 4.0 license or if the copyright holder’s requirements are incompatible with the CC BY 4.0 license, please either i) remove the figure or ii) supply a replacement figure that complies with the CC BY 4.0 license. Please check copyright information on all replacement figures and update the figure caption with source information. If applicable, please specify in the figure caption text when a figure is similar but not identical to the original image and is therefore for illustrative purposes only.

The following resources for replacing copyrighted map figures may be helpful:

USGS National Map Viewer (public domain): http://viewer.nationalmap.gov/viewer/

The Gateway to Astronaut Photography of Earth (public domain): http://eol.jsc.nasa.gov/sseop/clickmap/

Maps at the CIA (public domain): https://www.cia.gov/library/publications/the-world-factbook/index.html and https://www.cia.gov/library/publications/cia-maps-publications/index.html

NASA Earth Observatory (public domain): http://earthobservatory.nasa.gov/

Landsat: http://landsat.visibleearth.nasa.gov/

USGS EROS (Earth Resources Observatory and Science (EROS) Center) (public domain): http://eros.usgs.gov/#

Natural Earth (public domain): <http://www.naturalearthdata.com/>

**Response:** I have removed figure 1 from the manuscript.

Additional Editor Comments:

**Dear Author,**

I acknowledged author's work entitled: Burden and etiology of neonatal meningitis at Hawassa University Comprehensive Specialized Hospital. Even though, a single author the results illustrated the burden of meningitis among children*.* Therefore, this study will be useful for prevention and control of bacterial meningitis among children in the study area. The manuscript is more or less in line with the vision, objectives and instructions of PLoS One.

**Title:** ‘Burden and etiology of neonatal meningitis at Hawassa University Comprehensive Specialized Hospital’ It is better writer as, ‘Burden and bacterial etiology of neonatal meningitis at Hawassa University Comprehensive Specialized Hospital, Hawassa, Ethiopia’

**Abstract**

- Lines 9: ‘Introduction’ better replaced by ‘Background’.

**Response**: We greatly appreciate your valuable feedback and have made the necessary revisions accordingly. The manuscript now reflects all the corrections, which are clearly highlighted using the track change feature.

**Introduction**

- Line 82: …………………HUCSH, better written as, Hawassa University Comprehensive Specialized Hospital (HUCSH).

**Response:** Thank you for the comment. I have followed the suggested instructions and written the text accordingly.

**Materials and Methods**

- No sample size determination and sampling technique was described.

**Response:** We appreciate your feedback. The research conducted was a retrospective study covering a period of five years, from 2019 to 2023. The study encompassed all newborns who were admitted to the hospital during this timeframe and met the specified inclusion criteria. Since all newborns who visited HUCSH between 2019 and 2023 were considered in the study, we determined that a sample size calculation was unnecessary.

- Line 101: ‘Bacteria were identified using standard bacteriological techniques. Which bacteriological technique?

**Response:** We appreciate your comment. As this study is retrospective in nature, we acknowledge that we have no control over the laboratory method employed. This limitation is duly acknowledged in the discussion section of the study. Nevertheless, we have provided a concise overview of the sample collection, processing, and identification methods utilized within the hospital setting.

- Specimen collection and processing needs to be well written.

**Response:** Thank you for the comment. We have provided a concise description of the specimen collection method employed in the hospital. However, it is important to note that since this study is retrospective in nature, our involvement did not extend to specimen collection, processing, and bacterial identification.

- What standard microbiological procedures were employed in specimen collection, isolation, identification susceptibility testing and throughout the procedure?

**Response:** Thank you for the comment. In the case of suspected meningitis at HUCSH, a sample of 1 to 5 ml of cerebrospinal fluid (CSF) is collected from the patients and sent to the microbiology lab for further examination. Once in the lab, the CSF in the first tube is centrifuged and the resulting sediment is then inoculated onto culture media for analysis. However, it is important to note that in this particular study, these procedures were not carried out in their entirety due to its retrospective nature. Instead, a summary of the procedures that were performed has been included.

How could you standardize the suspension preparation during performing antimicrobial susceptibility testing?

**Response:** Your insight is greatly appreciated. Nevertheless, the manuscript does not present AST data as it may not be obligatory to describe the AST in the method section.

An ethical approval was also needed. What have you done for children positive for the microorganisms?

**Response**: We have uploaded ethical clearance during initial submission.

**Results**

- Table descriptions should be described well in terms of person, place and time.

**Response**: We have modified accordingly.

- Line 124: Table 2, words/phrases like: ‘CONS, GPR, NLF GNR’ requires legend or a full form as they appear for the first time.

Response: We have corrected accordingly

- Please follow the manuscript writing protocol of PLOS ONE for figure preparation and presentation.

**Response**: Thank you for suggestion, we have corrected the tables based on your suggestion.

- What is your base to classify the age group?

**Response**: Thank you for the comment. Bacteria transmission methods have been categorized by us. Meningitis that manifest in newborns under 7 days old are contracted vertically from the mother, while those occurring in infants older than 7 days are acquired horizontally from the hospital, family members, and healthcare workers.

- Why you missed to include the associated risk factors?

**Response**: Thank you for the comment. Due to the retrospective nature of this study, we were unable to obtain the necessary variables from the record. Our analysis focused on age group, sex, and year of admission to determine if there was any association with culture-confirmed meningitis. However, none of these variables showed a significant association. To provide further information, we have included chi-square values with their corresponding p-values in table 1.

- Authors tried to compare their findings with different reports from worldwide. They also better justify the reason for variation among results of different research findings with respect to theirs’ based on actual situation.

**Response:** Thank you for the comment. We have revised accordingly.

**Conclusions**

- Normally, conclusion emanates from the research.

**Response:** Our research revealed that the prevalence of culture-confirmed meningitis was significantly higher when compared to various studies carried out in Ethiopia and abroad. This observation was clearly reflected in our final analysis.

[Note: HTML markup is below. Please do not edit.]

Reviewers' comments:

Reviewer's Responses to Questions

**Comments to the Author**

1. Is the manuscript technically sound, and do the data support the conclusions?

The manuscript must describe a technically sound piece of scientific research with data that supports the conclusions. Experiments must have been conducted rigorously, with appropriate controls, replication, and sample sizes. The conclusions must be drawn appropriately based on the data presented.

Reviewer #1: Yes

Reviewer #2: Yes

2. Has the statistical analysis been performed appropriately and rigorously?

Reviewer #1: Yes

Reviewer #2: No

3. Have the authors made all data underlying the findings in their manuscript fully available?

The [PLOS Data policy](http://www.plosone.org/static/policies.action#sharing) requires authors to make all data underlying the findings described in their manuscript fully available without restriction, with rare exception (please refer to the Data Availability Statement in the manuscript PDF file). The data should be provided as part of the manuscript or its supporting information, or deposited to a public repository. For example, in addition to summary statistics, the data points behind means, medians and variance measures should be available. If there are restrictions on publicly sharing data—e.g. participant privacy or use of data from a third party—those must be specified.

Reviewer #1: Yes

Reviewer #2: Yes

4. Is the manuscript presented in an intelligible fashion and written in standard English?

PLOS ONE does not copyedit accepted manuscripts, so the language in submitted articles must be clear, correct, and unambiguous. Any typographical or grammatical errors should be corrected at revision, so please note any specific errors here.

Reviewer #1: Yes

Reviewer #2: Yes

5. Review Comments to the Author

Please use the space provided to explain your answers to the questions above. You may also include additional comments for the author, including concerns about dual publication, research ethics, or publication ethics. (Please upload your review as an attachment if it exceeds 20,000 characters)

Reviewer #1: The title "Burden and etiology of neonatal meningitis at Hawassa University Comprehensive
Specialized Hospital" needs modification" because fungal and viral agents are not considered throughout the manuscript. Simply modify the title, like the aim explained in the abstract section (L13–15).

**Response:** Thank you for the constructive comments; The title has been appropriately amended to reflect the necessary corrections. The manuscript, which includes all the modifications made, can be viewed with the track changes feature enabled.

Lines 13–15 and 16–18,  in the introduction and methods sections of the abstract, respectively, reveal different participant inclusions. A similar source population must be used, whereas all newborns less than 90 days of age and suspected newborns are explained, respectively.

**Response:** We have corrected accordingly.

Moreover, it is stated that "At HUCSH, isolation and identification of
19 bacteria were performed using standard microbiological techniques". Since it is retrospective, it must be explained in a different way.

**Response**: We agree with the comment, since this study a retrospective, samples were not collected and processed in this study. We have modified the phrase to show this.

In the results of the abstract section, "Line 1: The age of 767 (72.3%) was between 8 and 90 days" is unclear.

**Response**: It is modified to make it clear.

L52-53 "Among the various etiologies of meningitis, bacteria are responsible for the highest burden.
53 worldwide" is unrealistic. What about viruses?

**Response:** We share the comments raised by the reviewer. Viruses and fungus can also cause meningitis but they are not diagnosed in most cases. We have modified accordingly.

L69-71: "Death and neuropsychological sequelae from meningitis are higher in low-income countries because of a lack of facilities to manage the cases as early as possible." What about other very important factors?

**Response**: Thank you for the comment we have modified as ‘Death and neuropsychological sequelae from meningitis is higher in low-income countries because of a lack of diagnostic setup and facilities to manage the cases as early as possible’

Additionally, words like burden, magnitude, and prevalence are not used consistently at the beginning of your research question, and it is better to use only one of them throughout the document.

**Response**: Thank you for the pointing the inconsistency. Now it is corrected as ‘magnitude’

Consider L17 VS 83. Are you studying infants (L-89) or newborns?

**Response**: Thank you again to be consistent ‘infants’ is replaced with ‘newborns’

Line 91: silly mistake, the year (needs correction).

Response: Based on the comment it is corrected as ‘from 2019 to 2023’

Lots of information is missed in data collection, transportation, handling, and data quality control.

**Response:** As this study is retrospective, there is no way to control for sample collection, transportation, and handling. We have included sample collection and laboratory method used in the hospital. We have also included quality control for secondary data collection under subheading ‘data quality and analyses’

L130 to L132 "This life-threatening illness poses a significant risk, particularly in low-income countries, where it is associated with a high case fatality rate and long-term complications," needs rewriting (similar meaning by different phrases).

**Response**: It is modified.

Generally, there are some typos and grammar issues to accept after minor revision.

**Response**: All are checked and corrected.

Reviewer #2: General comments
The topic is very interesting and concerned with vulnerable study group. However the manuscript prepared as simple description. for example the author considered sex, age and the year of visit as independent variable but not tried to see the relationship between the outcome variable and independent variables. therefore much effort is needed to improve the manuscript.
specific comments

**Response**: Thank you very much for the comment. As this study is retrospective we did not able to get necessary variables from the laboratory record. We have checked for the age group, sex, and year of admission if they have association with culture-confirmed meningitis but none of them showed significant association. In table 1, we have added chi-square with p-value.

Abstract
Result:
#27-28 Coagulase- negative Staphylococci (CONS) were the most common agents of meningitis. (Please include the percentage and absolute no)

**Response**: It is corrected accordingly.

#29 Acinetobacter species (n=12.2; 13.3%) followed by Escherichia coli (n=9; 10%) and Klebsiella pneumonia. ‘n’ stands for absolute number, so it should be corrected

**Response**: It is corrected.

Conclusion:
#34-35 The overall magnitude of culture-confirmed meningitis was high. What is your based to say high? Have you compared with previous study in Ethiopia or any other country considering the confidence interval? Otherwise difficult to say high or low.

**Response**: Yes it is compared with studies conducted in Ethiopia and other countries. “The results of this study indicate a higher percentage compared to a study conducted in Addis Ababa, Ethiopia, which reported a rate of 4.7% (10), and Gondar, which reported a rate of 1.73% (11).”

Introduction
No overall data related to the burden were documented at national level. Please consider to include it. You may get it either from EDHS or from systematic review studies.

**Response**: Thank you for the comment we have searched but did not find with overall data related to burden of meningitis among newborns in Ethiopia.

Methods
Study design and area
Please mention the catchment area of the Hospital and the environmental factors that might be associated with meningitis.

**Response**: Additional information about the study site is included.

Please also write the detail of Hospital service and the number of clients seeking services.

**Response**: All requested information is included.

#91 from 2019 to 202. Kindly correct this figure

**Response**: It is corrected.

Study participants and data collection
Please briefly explain how the sumple collected and how the isolation and identification carried out.

**Response**: This is a retrospective study; it is difficult to have a control over the laboratory method used and this is mentioned as one of the limitation of study at the end of the discussion. A brief sample collection, processing, and identification used in the hospital was mentioned.

Data analysis and interpretation
Your study is simple descriptive. On the other hand your finding compared with other findings. Though you reported demographic characteristic, you didn’t associated your finding. Therefore I kindly revise your result accordingly.

**Response**: As this study is retrospective, it was difficult to get necessary variables from the record. Association of age group, sex, and year of admission with culture-confirmed meningitis was assessed; however, none of them showed significant association. In table 1, we have added chi-square with p-value.

It would be better if you show the trend of meningitis burden across the year too.

**Response**: It is shown in table 1

Discussion
The discussion part needs detail explanation, Justification needs scientific ground.

**Response**: The discussion is revised as shown in the manuscript with track change.

6. PLOS authors have the option to publish the peer review history of their article ([what does this mean?](https://journals.plos.org/plosone/s/editorial-and-peer-review-process#loc-peer-review-history)). If published, this will include your full peer review and any attached files.


If you choose “no”, your identity will remain anonymous but your review may still be made public.


**Do you want your identity to be public for this peer review?** For information about this choice, including consent withdrawal, please see our [Privacy Policy](https://www.plos.org/privacy-policy).

Reviewer #1: **Yes:** Zigale Hibstu

Reviewer #2: No

[NOTE: If reviewer comments were submitted as an attachment file, they will be attached to this email and accessible via the submission site. Please log into your account, locate the manuscript record, and check for the action link "View Attachments". If this link does not appear, there are no attachment files.]

While revising your submission, please upload your figure files to the Preflight Analysis and Conversion Engine (PACE) digital diagnostic tool, <https://pacev2.apexcovantage.com/>. PACE helps ensure that figures meet PLOS requirements. To use PACE, you must first register as a user. Registration is free. Then, login and navigate to the UPLOAD tab, where you will find detailed instructions on how to use the tool. If you encounter any issues or have any questions when using PACE, please email PLOS at [figures@plos.org](mailto:figures@plos.org). Please note that Supporting Information files do not need this step.
