# Supplementary material for: Burden and bacterial etiology of neonatal meningitis at Hawassa University Comprehensive Specialized Hospital, Hawassa, Ethiopia
Source: PLoS One. 2024 Aug 9;19(8):e0308523. doi: 10.1371/journal.pone.0308523 (PMC11315337; doi:10.1371/journal.pone.0308523)
Supplement: Attachment — Submitted filename: Response to Reviewers comments.docx [file pone.0308523.s003.docx]

**Response to reviewers and editor comments**

Additional Editor Comments:

- The author significantly improves the paper, still it requires a line by line through proofreading and make a correction in terms of punctuation and editorial issue.

**Response:** Thank you again, I have re-checked the manuscript and corrected issues related to punctuations and editorials as shown in the manuscript with track change.

- Title: Burden and etiology of neonatal meningitis at Hawassa University Comprehensive Specialized Hospital’ It is better written as, ‘Burden and bacterial etiology of neonatal meningitis at Hawassa University Comprehensive Specialized Hospital, Hawassa, Ethiopia.’ If you have any reason not to revise title you can raise the reason.

**Response**: Thank you for the comment; I have replaced the title with the suggested one. The change is shown in the manuscript with track change.

- Add some analysis techniques (Chi-square, P-value, etc) in the abstract as well as in the methods part.

**Response:** I have included chi-square and p-value as suggested in the abstract and method.

- Line 82: Study area design

**Response:** Thank you again for pointing the error, I have corrected it.

- Line 220: Data availability statement

**Response**: I have corrected as suggested.

[Note: HTML markup is below. Please do not edit.]

Reviewers' comments:

Reviewer's Responses to Questions

**Comments to the Author**

1. If the authors have adequately addressed your comments raised in a previous round of review and you feel that this manuscript is now acceptable for publication, you may indicate that here to bypass the “Comments to the Author” section, enter your conflict of interest statement in the “Confidential to Editor” section, and submit your "Accept" recommendation.

Reviewer #1: All comments have been addressed

Reviewer #2: All comments have been addressed

2. Is the manuscript technically sound, and do the data support the conclusions?

The manuscript must describe a technically sound piece of scientific research with data that supports the conclusions. Experiments must have been conducted rigorously, with appropriate controls, replication, and sample sizes. The conclusions must be drawn appropriately based on the data presented.

Reviewer #1: Yes

Reviewer #2: Yes

3. Has the statistical analysis been performed appropriately and rigorously?

Reviewer #1: Yes

Reviewer #2: Yes

4. Have the authors made all data underlying the findings in their manuscript fully available?

The [PLOS Data policy](http://www.plosone.org/static/policies.action#sharing) requires authors to make all data underlying the findings described in their manuscript fully available without restriction, with rare exception (please refer to the Data Availability Statement in the manuscript PDF file). The data should be provided as part of the manuscript or its supporting information, or deposited to a public repository. For example, in addition to summary statistics, the data points behind means, medians and variance measures should be available. If there are restrictions on publicly sharing data—e.g. participant privacy or use of data from a third party—those must be specified.

Reviewer #1: Yes

Reviewer #2: Yes

5. Is the manuscript presented in an intelligible fashion and written in standard English?

PLOS ONE does not copyedit accepted manuscripts, so the language in submitted articles must be clear, correct, and unambiguous. Any typographical or grammatical errors should be corrected at revision, so please note any specific errors here.

Reviewer #1: Yes

Reviewer #2: Yes

6. Review Comments to the Author

Please use the space provided to explain your answers to the questions above. You may also include additional comments for the author, including concerns about dual publication, research ethics, or publication ethics. (Please upload your review as an attachment if it exceeds 20,000 characters)

Reviewer #1: The authors have adequately addressed comments raised in a previous round of review the and I feel that this manuscript is now acceptable for publication. The manuscript technically soundful and the data support the conclusions. The manuscript is presented in an intelligible fashion and written in standard English.

**Response**: Thank you for checking and approving the change made.

Reviewer #2: Title
Line 1: Magnitude and Bacterial profile…….

**Response**: Thank you for the comment; I have corrected the title based on the editor comment.

Abstract

Line 13: Magnitude and Bacterial profile…….

**Response**: Thank you for the comment I have corrected accordingly

Introduction
Please make one paragraph on how the new borne acquire the infection

**Response**: I have included a paragraph on how the newborns acquire the infection.

Line 40-41: The sentence need citation

**Response**: I have included the citation as suggested.

Line 41-42: The sentence need citation

**Response**: I have included the citation as suggested.

Methods
Line 82: Study area desin – correct spelling error

**Response**: I have corrected it.

Line 85: The study time or period should be the first date of the date record up to the last date of data record.

**Response**: Thank you for the comment, I believe both times (time of data collection and admission to the hospital are important). I modified to indicate both time intervals as “Data collection period was from14/10/2023 to 14/11/2023. Data was collected from patients who were admitted to HUCSH within time interval of 2019 to 2023.”

Line 86: south of Addis Ababa, capital City of Ethiopia

**Response**: I have corrected accordingly.

Results
Line 138: age group of 0 to 8 days, correct the range

**Response**: I have corrected it.

Line 132-133: The magnitude of culture-confirmed meningitis was high among females and among those who were within the age range of 8 to 90 days. However your analysis indicated the p value is greater than 0.05 (0.18). is it possible to higher or lower? Please recheck it?

**Response:** Thank you for the comment. The proportion is higher among females (9.8% vs 7.5%) and among those who were within the age range of 8 to 90 days (9.5% vs 5.8%) but these differences are not statistically significant as p value in both cases is greater than the cut point which 0.05

7. PLOS authors have the option to publish the peer review history of their article ([what does this mean?](https://journals.plos.org/plosone/s/editorial-and-peer-review-process#loc-peer-review-history)). If published, this will include your full peer review and any attached files.


If you choose “no”, your identity will remain anonymous but your review may still be made public.


**Do you want your identity to be public for this peer review?** For information about this choice, including consent withdrawal, please see our [Privacy Policy](https://www.plos.org/privacy-policy).

Reviewer #1: **Yes:** Zigale Hibstu Teffera

Reviewer #2: No

[NOTE: If reviewer comments were submitted as an attachment file, they will be attached to this email and accessible via the submission site. Please log into your account, locate the manuscript record, and check for the action link "View Attachments". If this link does not appear, there are no attachment files.]

While revising your submission, please upload your figure files to the Preflight Analysis and Conversion Engine (PACE) digital diagnostic tool, <https://pacev2.apexcovantage.com/>. PACE helps ensure that figures meet PLOS requirements. To use PACE, you must first register as a user. Registration is free. Then, login and navigate to the UPLOAD tab, where you will find detailed instructions on how to use the tool. If you encounter any issues or have any questions when using PACE, please email PLOS at [figures@plos.org](mailto:figures@plos.org). Please note that Supporting Information files do not need this step.
